# A Campaign for Food Economy

**Published:** 1917-09-15

**Authors:** 


### The Hospital

The Workers' Newspaper of Administrative Medicine and Institutional Life, Administration, National Insurance and Health.

No. 1632, Vol. LXII. SATURDAY, SEPTEMBER 15, 1917. PRICE ONE PENNY

### A CAMPAIGN FOR FOOD ECONOMY.

The British public has often been urged to arrange its food habits in accordance with the precepts of authority and to abandon in this regard the promptings of instinct and of tradition. Learned men have produced rules and data which they have discovered in the laboratory, economists have arranged tables of values both formidable and impressive, and cranks and faddists have sounded alarms and guaranteed delights each according to his peculiar creed. On hardly any other topic, save perhaps religion and politics, has there been so zealous a display of persevering preaching. Yet the great mass of the public has remainder unmoved. Custom and appetite have continued as guiding lights, and the preachers and their pamphlets have prophesied in vain. This dull resistance to instruction has caused much distress in circles where superior persons are wont to resort, but for our own part we have always held that such an attitude is largely determined by an unconscious wisdom born of the inherited experiences of countless generations. Man had learned his food lessons long before the physiologist appeared on the scene, and while the latter may usefully explain our habits he is hardly competent to prescribe them.

Nowadays, however, fate has brought us a new teacher, and he speaks with an authority professing a very forcible appeal. Economy, both individual and national, is in existing circumstances a concern of most of us, and it takes on a dignity which in smoother times was often denied to it. Patriotism recognises it as a duty, and the pressure of the hour largely compels it as a necessity. Hence we are disposed gladly to listen to the food economist, though we had no ready ear for the chemist and the crank. Behind the teaching stands the dread sanction of the submarine, and the risk of national disaster. The change is an enormous one, and is realised with difficulty in a country where for long food has been both abundant and cheap. Yet the situation has to be faced, and no easy optimism will conjure away the peril. We may exercise if we will habits of wastefulness and of inefficiency in times of peace, but with war at our doors such habits become accomplices of the enemy. Now, at all events, we must pay heed to our food supplies and to the methods of using them, and it is not to be doubted that in this direction a large contribution is to be secured for the success of the national cause. This conviction is written in large letters by our governing authorities, as the strange figure of a Food Controller clearly shows. But while the official direction and control of a Government Department can do much, and have done much, the essential issue lies in the hands of the people themselves. It is in the individual home and by the individual housekeeper that the question will be finally determined, and it is here that the lessons of food economy need to be carried and enforced. Admittedly there is much need of knowledge if the best results are to be obtained, and any effort to supply this knowledge claims the sympathy and good will of all persons who love their country and wish to see the full triumph of the national cause. There is at our gates a determined and unscrupulous enemy, whose one hope of success is the paralysis of our food supplies. To defeat this attempt we have mainly to rely on ourselves, and we are assured on the highest authority that if only we cultivate economy our safety is secure. Food regulations having legal force have their claim and value; our seamen may continue, as doubtless they will continue, cheerfully to face all risks to bring their ships into port; and our orators and masters will not cease in their patriotic appeals and exhortations. But not any one of these, nor the whole of them, will mean success unless the individual citizen realises his duty and performs it.

This duty, however, needs direction and intelligence. A sort of semi-starvation is not the ideal at which to aim, for this would be fatal both to the will and to the working power of the nation. What is needed is a recognition of the fact that the supply of food is a limited one, and that the problem is to utilise this limited quantity in the most efficient manner. The limitation may be imposed by authority, and the range of this is likely to become more rather than less extensive. But wise employment can only be secured by voluntary effort in combination with knowledge. Here it is that the individual becomes the unit of importance and that instruction may make effective personal desire and good will. Outside authority may succeed in cutting down the quantity of food we may be permitted to obtain, but the problem of a wise use of this quantity must be solved in the individual household. The first of these tasks, as we all recognise, has been undertaken in the most unselfish and patriotic spirit by Lord Rhondda. It is not unwelcome to learn that to the success of the equally important home economies Lady Rhondda has determined to contribute her help and influence. Her ladyship has issued an appeal for £5,000 to ensure the success of a food economy campaign instituted by the National Food Reform Association. This Association has made a special study of foods from the economical standpoint, and it seeks to spread the knowledge it has acquired by many missionary enterprises. It is ready to contribute help and advice to all the organisations which are striving in the present emergency to economise the food supplies and to use them to the greatest advantage. Never more than now has knowledge of this order been so useful in the homes of the people, and it is because Lady Rhondda realises this that she makes her appeal for co-operation and support. There is wisdom as well as patriotism in the undertaking, and it sets out under the best auspices. We have pleasure in commending it to the effective sympathy of our readers, and will gladly forward to her ladyship any contributions committed to our care.

